# The Influence Law and Etching Verification of Sub-Wavelength Longitudinal Field Generation

**DOI:** 10.3390/nano16201300

**Published:** 2026-10-14

**Authors:** Ji Wang, Kaiwen Ding, Wei Guo, Lin Li

**Affiliations:** 1 Research Center for Laser Extreme Manufacturing, Ningbo Institute of Materials Technology & Engineering, Chinese Academy of Sciences, Ningbo 315201, China; 2 Ningbo College of Materials Technology & Engineering, University of Chinese Academy of Sciences, Ningbo 315201, China; 3 Zhejiang Key Laboratory of Laser Extreme Manufacturing for Difficult-to-Machine Materials, Ningbo Institute of Materials Technology & Engineering, Ningbo 315201, China

**Keywords:** femtosecond laser, nanoscale fabrication, sub-diffraction limit, longitudinal field, optical field modulation, laser processing

## Abstract

The generation of sub-diffraction-limit focal spots is essential for nanoscale manufacturing, and tight focusing of radially polarized beams provides a promising route to achieve this goal through the longitudinal field. This work proposes an annular-modulation-enhanced focusing method that combines numerical aperture (NA) optimization, annular ring width control, and Bessel–Gaussian beam parameter tuning to enhance the longitudinal field and compress the focal spot. The method selectively transmits high-angle components that contribute dominantly to the longitudinal field, while filtering out low-angle components that broaden the focal spot. The physical boundary of the effective regulation window was identified through parameter space mapping. By optimizing the parameters, the simulated longitudinal field content exceeded 85%, and the focal spot size was reduced below a 0.2 wavelength (λ). Moreover, under far-field conditions, etching was performed on single-crystal silicon to achieve a straight line with a width of approximately 254 nm, corresponding to approximately λ/4. These results indicate that Bessel–Gaussian beam annular modulation can not only achieve sub-wavelength spots but also has excellent potential in the field of nanomanufacturing.

## 1. Introduction

Nanofabrication has become a core driving force in modern science and technology, with applications spanning semiconductor devices, photonic integration, and biomedical engineering [1]. As device feature sizes continue to shrink toward the nanometer scale, traditional optical processing methods face fundamental constraints imposed by the diffraction limit. According to Abbe diffraction theory, the lateral dimension of a focused spot cannot be smaller than half the wavelength, which fundamentally limits the spatial resolution of optical methods in nanofabrication [2]. Therefore, achieving sub-diffraction focal spots under far-field conditions has become a critical scientific challenge in nanofabrication.

To overcome this limitation, researchers have developed various laser nanofabrication techniques. Two-photon lithography (TPL) exploits the nonlinear absorption of femtosecond lasers [3,4]. Photopolymerization only occurs at the center of the focal point, achieving sub-diffractive three-dimensional nanostructures [5]. Stimulated emission depletion (STED) lithography introduces a doughnut-shaped depletion beam [6,7]. This beam suppresses the aggregation reaction in the outer region of the excitation point, thus reducing the feature size to several tens of nanometers [8]. However, STED imposes strict requirements on photoresist systems, requiring complex dual-beam alignment. Surface plasmon polariton (SPP) near-field lithography utilizes localized field enhancement from metallic nanostructures [9,10]. Its working distance is restricted to the near-field region. Laser-induced periodic surface structures (LIPSS) can be generated in the far field. These structures arise from the interaction between laser radiation and microscopically rough material surfaces, producing periodic nanoripples [11]. The period of the nanoripples is unstable, and their extension direction is typically perpendicular to the polarization direction [12]. Super-oscillatory lenses achieve far-field sub-diffraction focusing through multi-ring phase modulation, with lateral dimensions of 0.34λ to 0.42λ, but sidelobe suppression remains a challenge [13,14]. Microsphere nanoscopy leverages the near-field magnification effect of microspheres to achieve a linewidth of 75 nm, but the contact-mode operation limits its application range [15]. While each technique offers distinct advantages, a common challenge persists in simultaneously achieving sub-wavelength spot sizes and sufficient energy density under far-field conditions.

Among the various approaches to breaking the diffraction limit, the generation and enhancement of the longitudinal electric field have attracted considerable attention. Tight focusing of radially polarized beams is a well-established approach for generating a longitudinal electric field. In a radially polarized beam, the polarization direction is oriented radially with respect to the optical axis across the beam cross-section. The Richards–Wolf vectorial diffraction theory provides a rigorous theoretical framework for describing this focusing process [16,17]. Studies have shown that the focal spot size of the longitudinal component after special light field modulation is significantly smaller than that of the transverse component, with highly localized energy distribution, offering a promising route to sub-wavelength energy deposition under far-field conditions [18,19,20,21,22]. A longitudinal polarized light needle with a diameter of 0.43λ was created by modulating the energy field of five annular rings using binary optics [23]. This effect has been verified in applications including particle acceleration, optical trapping, and laser ablation [24,25,26,27,28].

However, existing studies face several key limitations. First, most works focus on a single modulation approach, lacking systematic investigation of multi-parameter combined modulation. Second, the influence of the intrinsic mode parameters of the incident beam is not well understood. The mechanism of the combination of a Bessel–Gaussian beam with NA and ring modulation has not been quantitatively revealed [29]. Third, the effective regulation window for longitudinal field enhancement remains unclear.

To address these issues, this work innovatively proposes the Bessel–Gaussian Annular Modulation (BGAM) method, which differs from the multi-ring strategy based on binary optics. BGAM systematically reveals the evolution law of longitudinal field content and focal spot compression limit through the synergistic effect of NA optimization, annular modulation, and the Bessel–Gaussian beam coefficient. This work establishes a three-dimensional parameter space mapping among NA, ring width ratio, and β, achieving a longitudinal field content exceeding 80% and a focal spot full width at half maximum below 0.2λ. It is noteworthy that this method etched grooves with a width of approximately 254 nm on single-crystal silicon, preliminarily demonstrating the potential of this method in sub-diffraction limit processing.

## 2. Methods and Equipment

### 2.1. Methods

**Figure 1:**
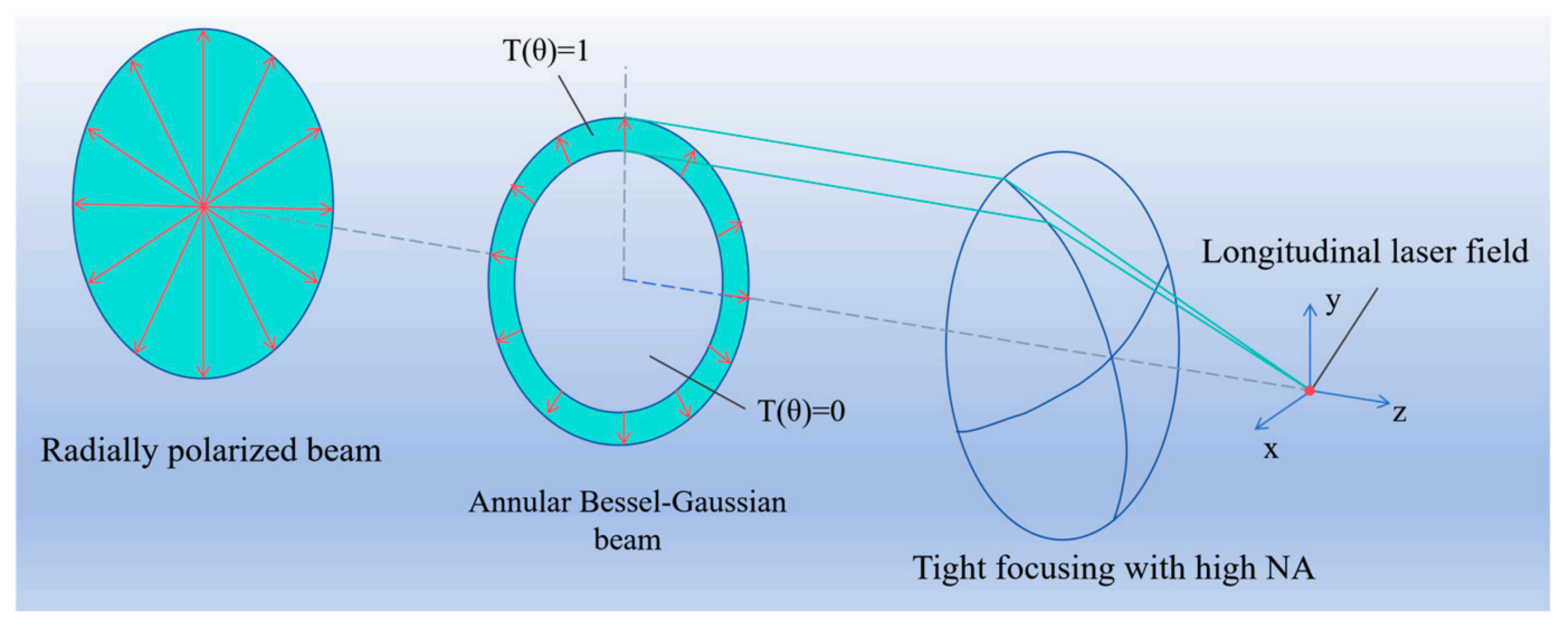
Schematic diagram of the sub-wavelength longitudinal field laser generation method. Radially polarized beam, annular beam modulation and tight focusing. The red arrow indicates the polarization direction of the electric field on the spot plane at that location.

The experiment used a femtosecond laser (FemtoA-IR-10-10, Huaray, Wuhan, China) in the near-infrared wavelength band. The core parameters of the laser system are summarized in Table 1. In this article, we propose the BGAM method to obtain a longitudinal field laser in a local field, achieving the sub-diffraction limit. As shown in Figure 1, the laser beam passed through a vector beam converter (VR1-1030, LBTEK, Wuhan, China), which transformed the linearly polarized Gaussian beam into a radially polarized beam. In a radially polarized beam, the polarization direction is oriented radially with respect to the optical axis across the beam cross-section. Then, the radially polarized beam undergoes Bessel annular modulation through an axicon lens, with the central area completely obscured and the peripheral area producing a fully transparent effect. This configuration generated a radially polarized Bessel–Gaussian beam with an annular intensity distribution. Finally, the modulated beam was focused by a high-NA objective lens (CFI Plan Apo Lambda 60XC, Nikon, Tokyo, Japan). Due to the tight focusing effect of the objective lens, a longitudinal electric field component emerged at the focal plane, with the polarization direction aligned along the propagation axis. A three-dimensional nano-positioning stage (FS-1050UPX, OptiSigma, Tokyo, Japan) with a maximum scanning speed of 5 mm/s and positioning accuracy of 1 nm was used for sample positioning.

### 2.2. Characterization and Materials

A beam profiler was used to record the spatial intensity distribution of the spot. Single-crystal silicon wafers with polished surfaces were selected as the substrate material. The silicon wafer was fixed onto the nano-positioning stage. After laser processing, the sample was cleaned with anhydrous alcohol and dried naturally. Scanning electron microscopy (SEM, MAGNA, TESCAN, Brno City, Czech Republic) was used to measure the processing morphology of the sample surface.

## 3. Results

### 3.1. Modulation Mechanism of Longitudinal Field Laser

According to the Richards–Wolf vectorial diffraction theory [14,15], the electric field distribution of a tightly focused radially polarized beam in the focal region can be expressed as Equations (1) and (2):

(1) $$E_{z}\left(\theta ,z\right)=\mathrm{iA}\int_{0}^{\alpha }\cos^{1/2}\theta \times \sin^{2}\theta \times l\left(\theta \right)\times T\left(\theta \right)\times J_{0}\left(\mathrm{krsin\theta}\right)\times e^{\mathrm{ikzcos\theta}}\mathrm{d\theta},$$

(2) $$E_{r}\left(\theta ,z\right)=A\int_{0}^{\alpha }\cos^{1/2}\theta \times \mathrm{sin\theta}\times \mathrm{cos\theta}\times l\left(\theta \right){\times T\left(\theta \right)\times J}_{1}\left(\mathrm{krsin\theta}\right)\times e^{\mathrm{ikzcos\theta}}\mathrm{d\theta},$$

Here, *E_z_*(*θ*,*z*) and *E_r_*(*θ*,*z*) represent the radial and longitudinal field laser at the focal plane, respectively. Parameter *A* is the amplitude of the field intensity, which is a dimensionless parameter. *α* = *arcsin*(*NA*) is the maximum convergence angle, k=2πλ is the wavenumber, and *J*_0_ and *J*_1_ are the zeroth and first-order Bessel functions of the first kind. The function *l*(*θ*) describes the amplitude distribution of the incident Bessel–Gaussian beam as Equation (3):

(3) $$l\left(\theta \right)=e^{-\beta \frac{\mathrm{sin\theta}}{\mathrm{sin\alpha}}}\times J_{1}\left(2\beta \frac{\mathrm{sin\theta}}{\mathrm{sin\alpha}}\right),$$

Here, *β* denotes the ring parameter of the Bessel–Gaussian beam, which controls the beam profile. The annular modulation function *T*(*θ*) is defined as Equation (4):

(4) $$T\left(\theta \right)=\left\{\begin{array}{l} 0,\;\mathrm{for}\;0<\theta <r_{1}\\ 1,\;\mathrm{for}\;r_{1}\ll \theta \ll R \end{array}\right.,$$

Here, *r*_1_ is the inner radius of the annular beam. *R* is the radius of the outer annular beam, with a value of R=sinαNA. The amplitude transmittance of the inner annular region is 0, achieving complete occlusion. The amplitude transmittance of the outer annular region is 1.

### 3.2. Effect of NA on Sub-Wavelength Longitudinal Field Laser Generation

First, the influence of NA on the generation of a longitudinal field was investigated by comparing two NA values of 0.75 and 0.95. At the focal plane (z = 0), the longitudinal field laser exhibited a bright central spot accompanied by first-order diffraction rings at radial distances of 1 to 1.5 wavelengths. The energy of these rings was substantially lower than that of the central spot. In contrast, the transverse field laser displayed an annular intensity distribution resembling a doughnut shape, with zero intensity at the center. The composite field laser, representing the vector superposition of the longitudinal and transverse components, exhibited distinct patterns at different NA values.

**Figure 2:**
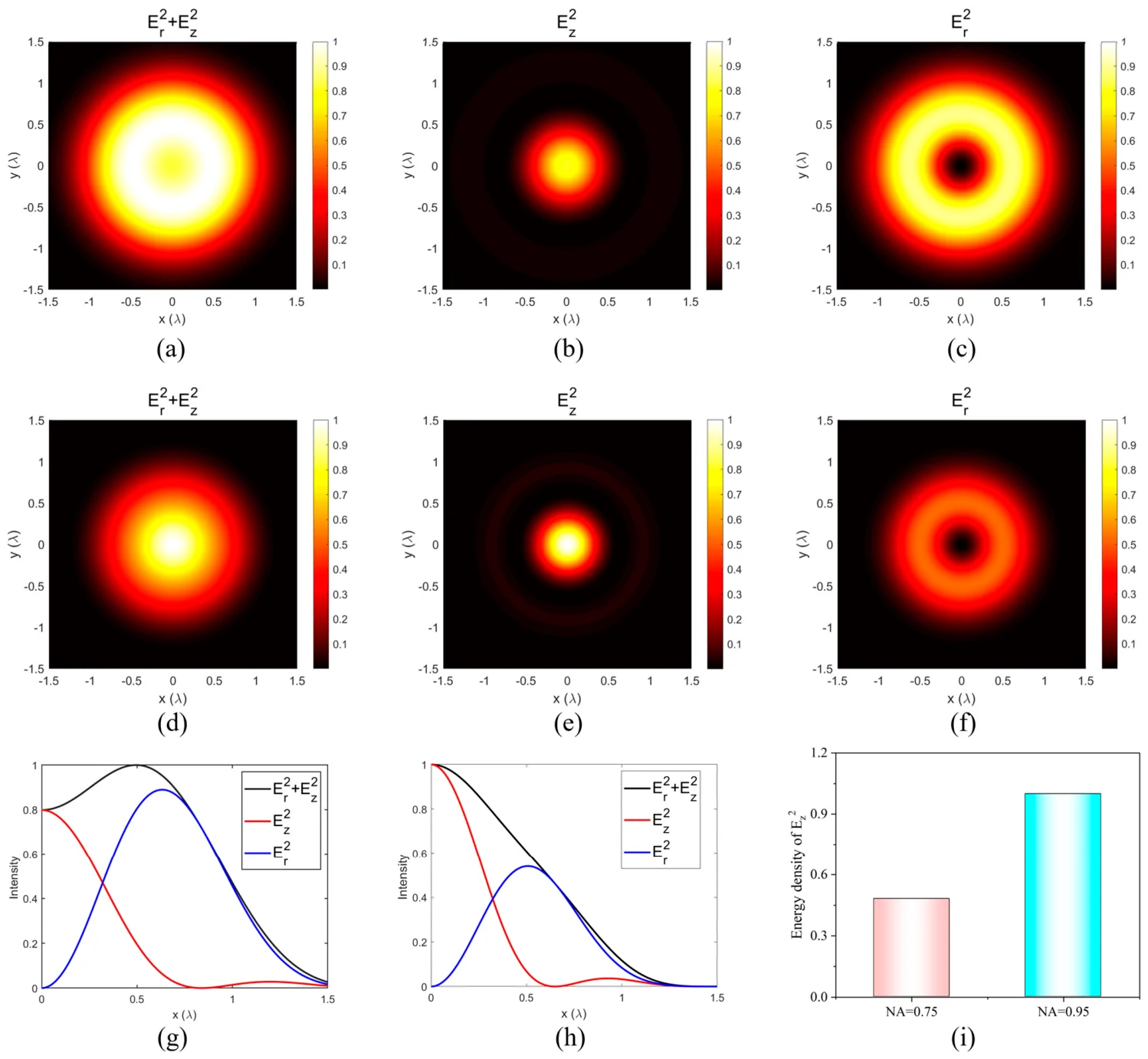
Simulated energy distributions of focal fields at different NAs. (**a**–**c**): 2D energy field color map of composite, longitudinal, and transverse field laser under an NA of 0.75. (**d**–**f**): 2D energy field color map of composite, longitudinal, and transverse field laser under an NA of 0.95. (**g**,**h**) Cross-sectional profiles of composite, longitudinal, and transverse fields at an NA of 0.75 and 0.95, respectively. (**i**) Energy density of E_z_^2^ comparison between an NA of 0.75 and 0.95.

Under an NA of 0.75, the composite field laser displayed a crater-like profile with lower intensity at the center and higher intensity in the periphery. This indicates that the transverse field component still dominated over the composite field laser. However, for an NA of 0.95, the composite field laser exhibited a Gaussian-like profile with maximum intensity at the center. This demonstrates that increasing the NA from 0.75 to 0.95 enables a crossover from transverse-field-dominated focusing to longitudinal-field-dominated focusing. At an NA of 0.75, the amplitude of the longitudinal field laser is slightly lower than that of the transverse field, which is the threshold at which the longitudinal field laser begins to generate (Figure 2g). The relative amplitude of the longitudinal field increased from 0.80 to 1.0 when the NA was raised from 0.75 to 0.95, while the relative amplitude of the transverse field decreased from 0.90 to 0.55. At this point, a strong longitudinal field characteristic is displayed at z = 0.

From the perspective of focal spot size, a higher NA (0.95) was found to be advantageous for reducing the FWHM of the longitudinal component. The FWHM of the composite field decreased from 0.981λ (NA = 0.75) under transverse-field-dominated conditions to 0.611 λ under longitudinal-field-dominated conditions (NA = 0.95). This reduction is attributed to the field enhancement effect of the longitudinal component, which effectively compresses the focal spot. The peak energy density increased by approximately a factor of two when the longitudinal field became dominant (Figure 2i). This enhancement is particularly beneficial for material processing applications.

### 3.3. Effect of Bessel Annular Modulation on Spot Size

**Figure 3:**
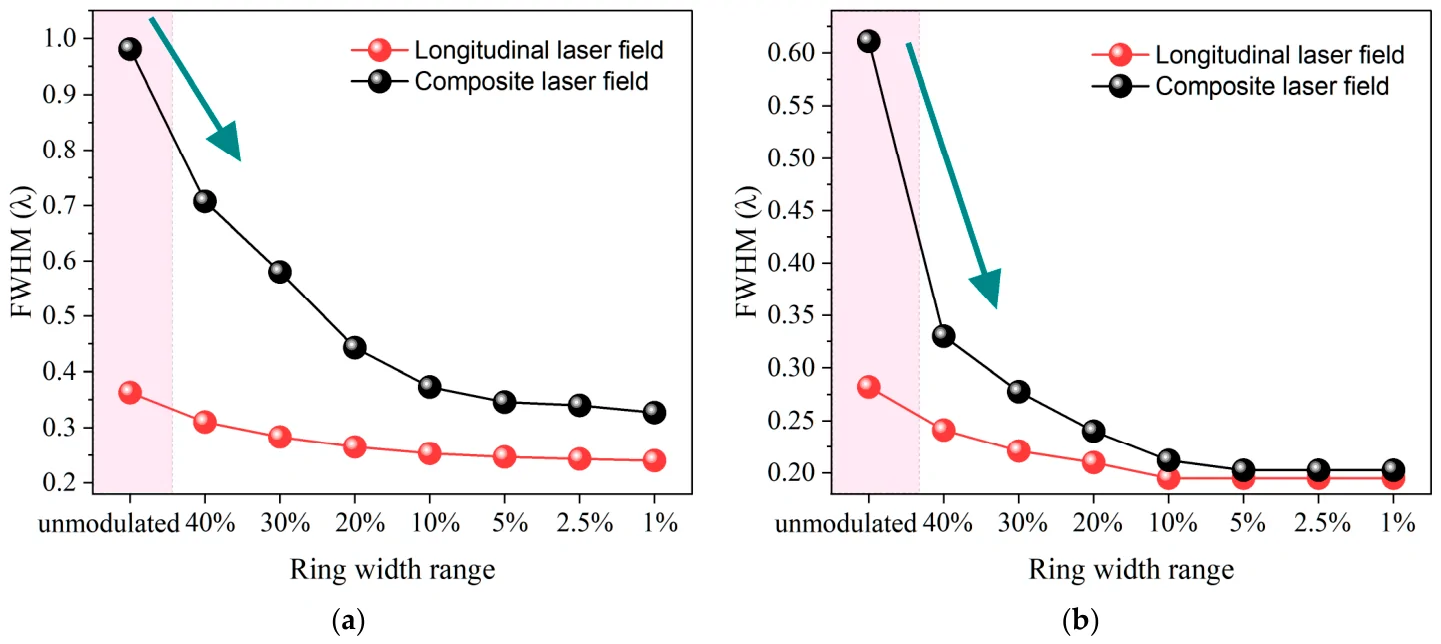
Comparing the FWHM trends of annular Bessel–Gaussian modulation and unmodulated: (**a**) NA = 0.75: FWHM trends for composite laser field and longitudinal component as a function of ring width. (**b**) NA = 0.95: FWHM trends under the same conditions. The red highlighted zone represents the incident beam without annular Bessel–Gaussian modulation.

After establishing the role of NA, the effect of annular modulation on the focal spot size was investigated. The annular modulation was implemented according to Equation (4), where the transmission function within the inner radius, *r*, is set to 0. The normalized ring width, ∆*r*, was used as the horizontal axis in Figure 3, represented by Equation (5).

(5) $$∆r=\frac{R-r_{1}}{R}\times 100\%,$$

The full-width-at-half-maximum (FWHM) results of the longitudinal and composite fields obtained from simulation calculations are shown in Figure 3. The total field exhibited a faster decay rate compared to the longitudinal field, which can be attributed to the rapid suppression of the radial component under annular modulation. Without annular modulation, the FWHM of the composite field is 0.981λ (Figure 2g). The FWHM of the composite field begins to decrease with annular modulation. At a ring width of 40%, the FWHM is reduced to 0.707λ, which still does not surpass the diffraction limit. For the longitudinal field, annular modulation produces the same trend. Without modulation, the FWHM of the longitudinal field is 0.363λ. Correspondingly, at a ring width of 40%, the FWHM is 0.310λ. When the ring width decreases to 10%, the FWHM is reduced to 0.253λ. The FWHM of the longitudinal field decreases monotonically with a decreasing ring width. Then, the longitudinal field FWHM reached 0.240*λ* at a ring width of 1%, thus surpassing the optical diffraction limit (Figure 3a). This demonstrates that Bessel annular modulation can overcome the inherent limitation of low-NA focusing where the transverse field dominates. This is a more effective longitudinal field enhancement strategy than increasing the NA.

The physical mechanism underlying the longitudinal field enhancement can be understood from the Richards–Wolf integral. The longitudinal field component *E_z_*(*θ*,*z*) is proportional to sin^2^θ. Table 2 presents the weighting of the spatial frequency factor sin^2^θ in the Richards–Wolf integral for the longitudinal field. At small angles near the pupil center, sin^2^θ approaches zero, indicating that these low-angle components contribute negligibly to *E_z_*(*θ*,*z*). At large angles near the pupil edge, sin^2^θ reaches its maximum, indicating that these high-angle components are the dominant contributors to *E_z_*(*θ*,*z*). From a spatial perspective, only the high-frequency components at the pupil edge contribute significantly. The annular aperture blocks the central low-angle region (low-frequency components) while transmitting the peripheral high-angle region (high-frequency components). In essence, it removes the portion that contributes nothing to the longitudinal field and preserves the portion that contributes most. A high NA allows high-frequency components with larger convergence angles to participate in focusing, which amplifies the sin^2^θ weighting factor in the integral. The annular modulation further amplifies this effect by filtering out the low-frequency components. Therefore, the BGAM method functions by filtering out low-frequency components while retaining high-frequency components, thereby selectively enhancing the longitudinal field content.

Moreover, at an NA of 0.95, the longitudinal field FWHM was further reduced to 0.195*λ* at a 10% ring width (Figure 3b). This confirms that high NA and annular modulation act synergistically to enhance the longitudinal field. However, unlike the low-NA case, the longitudinal FWHM at NA = 0.95 did not continue decreasing when the ring width was narrowed from 10% to 1%. This saturation behavior indicates that the annular modulation has an optimal range (corresponding to ∆*r* ranging from 5% to 10%) beyond which further narrowing yields diminishing returns.

**Figure 4:**
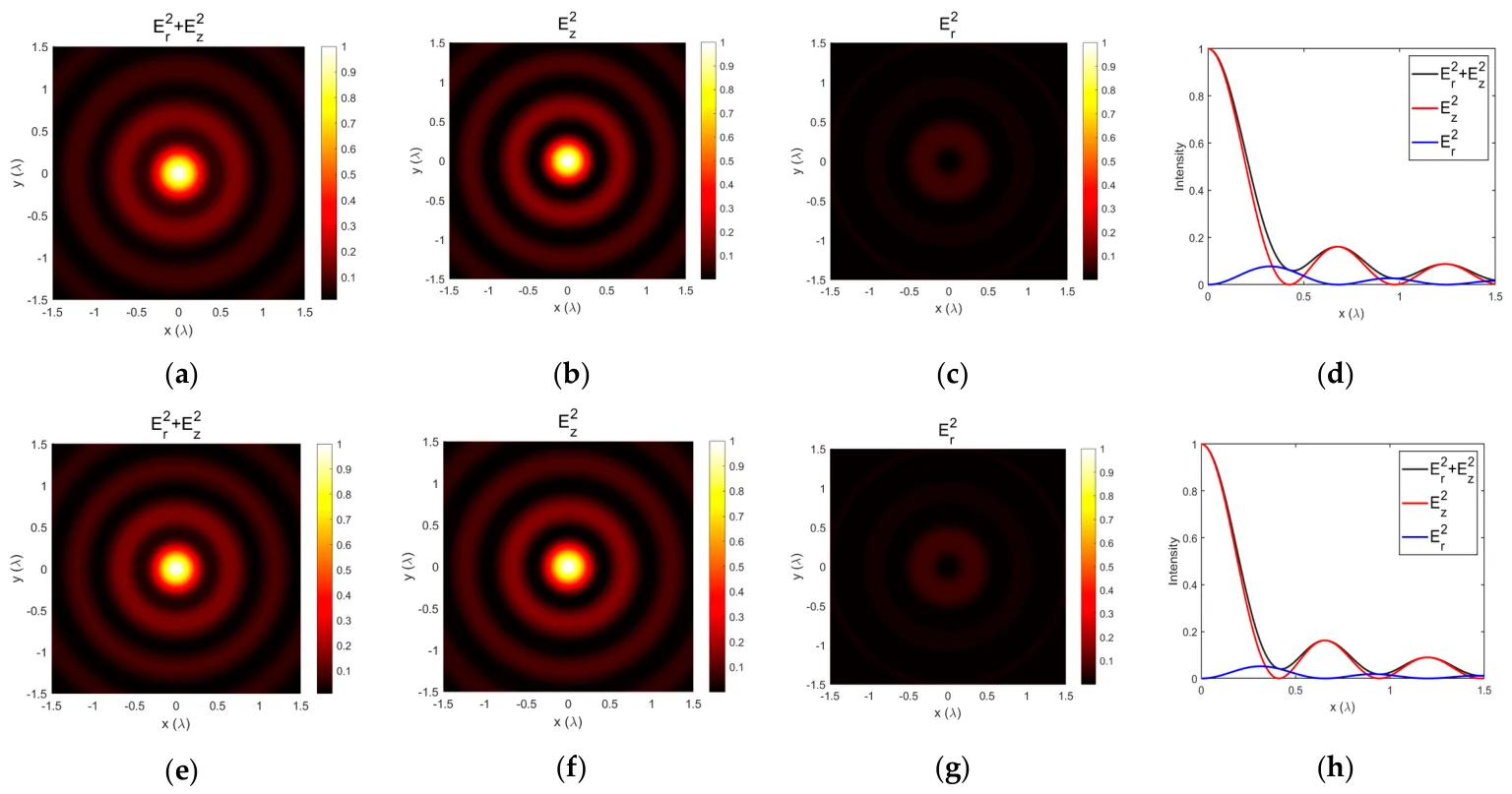
2D energy distributions at NA = 0.95: (**a**–**d**) ring width of ∆*r* = 10%. (**e**–**h**) ring width of ∆*r* = 1%. Composite, longitudinal, and transverse laser fields are shown for each case.

Furthermore, Figure 4 presents the 2D energy distributions of the beam at ring widths of 1% and 10%. The simulation results for both cases were nearly identical in terms of spatial energy distribution and radial intensity profiles. This confirms that further narrowing the ring width from 10% to 1% does not provide additional improvement in the longitudinal field distribution. The center of the longitudinal field presents a sub-wavelength bright spot, accompanied by a low peak energy (normalized intensity of 0.1–0.2) multi-order diffraction ring. The center of the transverse field is the dark spot, with a lower peak energy than the longitudinal diffraction ring. The composite field is almost entirely dominated by the longitudinal field in terms of both energy distribution and spot size. The normalized strength of the first-order longitudinal field remains unchanged under annular modulation with ring widths of 10% and 1%. However, the normalized intensity of the first-order transverse field under a ring width of 10% is 0.08, slightly higher than 0.05 under a ring width of 1% (Figure 4d,h). Although this does not affect the longitudinal characteristics of the light spot, it has a slight impact on the adjustment of the longitudinal field content in space (Figure 7).

Considering the practical difficulties in fabricating extremely narrow annular apertures, a ring width of 10% represents the optimal parameter that balances modulation effectiveness and experimental feasibility. The limiting longitudinal field FWHM achievable with a single annular ring mask is 0.195λ.

### 3.4. Effect of Bessel–Gaussian Beam Coefficient β

**Figure 5:**
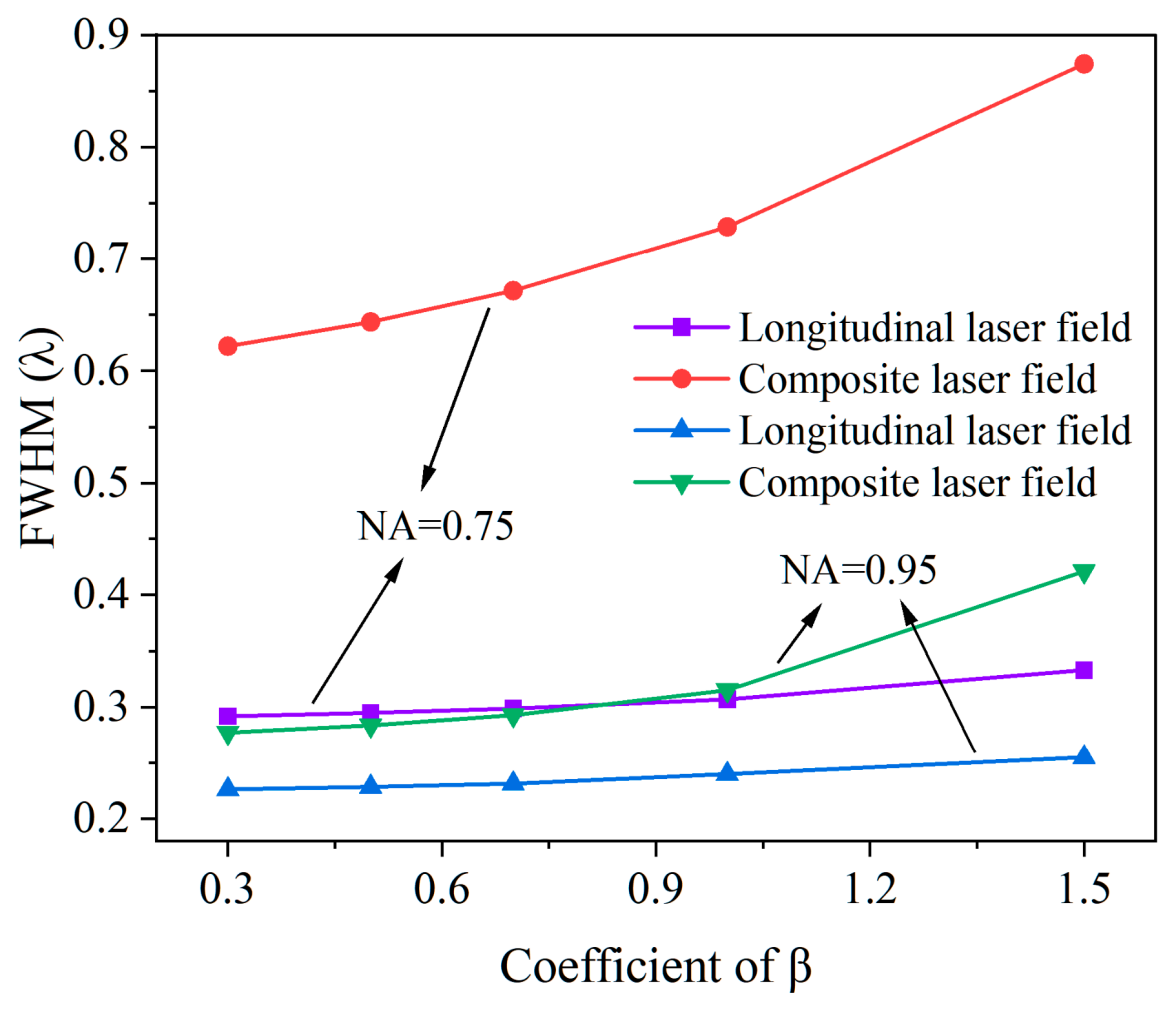
The influence trend of β on the FWHM of the longitudinal and composite laser fields without annular modulation.

**Figure 6:**
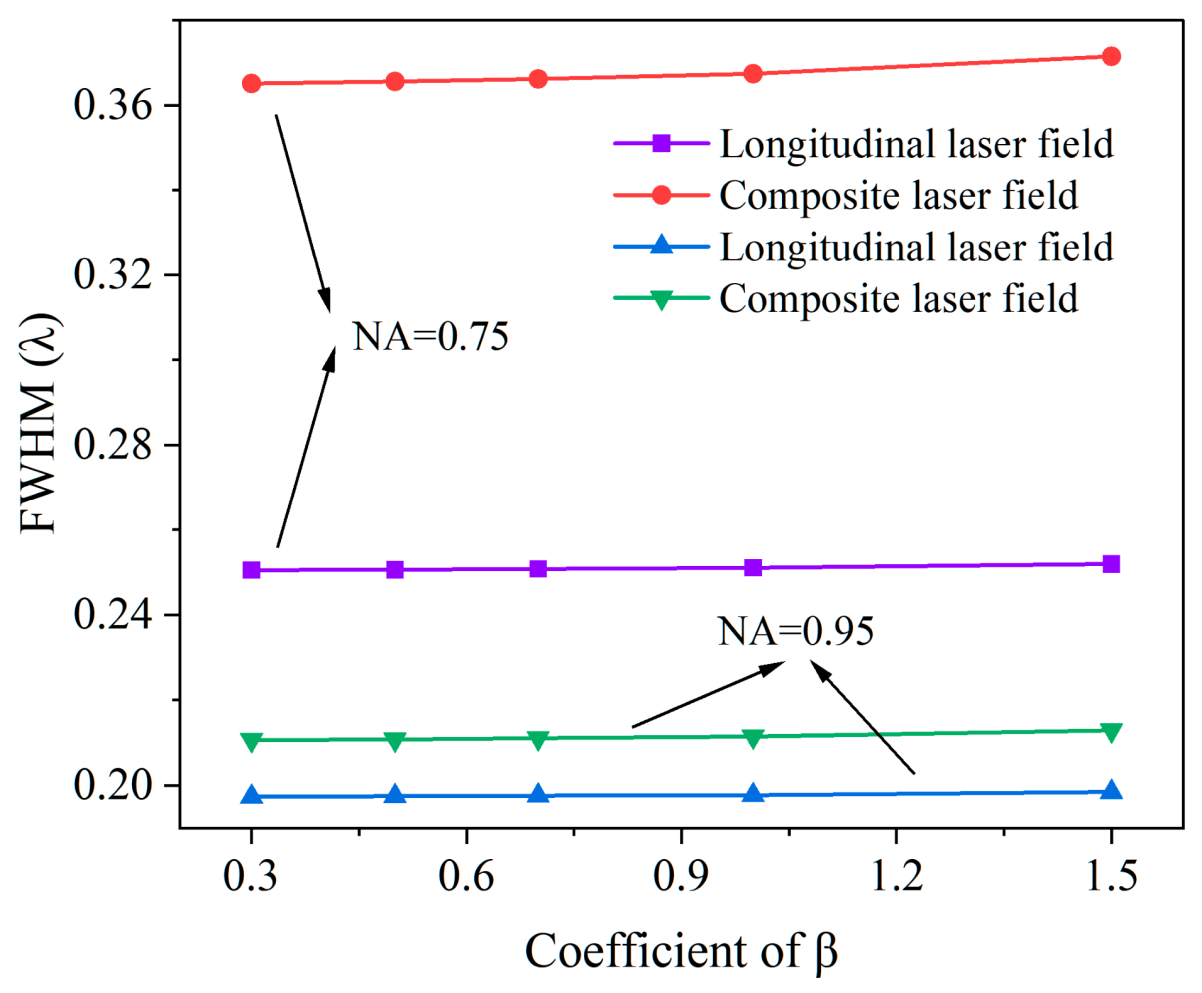
The influence trend of β on the FWHM of the longitudinal and composite laser fields under annular modulation with ∆*r* = 10%.

As shown in Equations (2) and (3), the focal field distribution at z = 0 is also governed by the coefficient β, which characterizes the amplitude distribution of the incident Bessel–Gaussian beam. The beam profile is determined by the competition between the exponential decay term exp(−β∙sinθ/sinα) and the Bessel oscillation term J_1_(2β∙sinθ/sinα). Table 3 summarizes the beam profile characteristics for the β values considered in this work. For a small β, the exponential decay is slow, and the beam energy extends to larger angles. As β increases, the exponential decay, exp(−β∙sinθ/sinα), becomes increasingly dominant, suppressing the energy at large angles and shifting the beam energy toward smaller angles. Since the longitudinal field is proportional to sin^2^θ and derives primarily from the high-angle components, this energy shift reduces the longitudinal field contribution (Figure 5 and Figure 6).

Figure 5 shows the effect of the coefficient β on the longitudinal field FWHM under circular illumination (without annular modulation). At NA = 0.75, the increase in β leads to an increase in the longitudinal FWHM from 0.307λ (β of 1.5) to 0.333λ (β of 1.0). At NA = 0.95, the longitudinal FWHM only slightly increased from 0.240λ (β of 1.5) to 0.255λ (β of 1.0) under the same coefficient variation. These results demonstrate that the longitudinal field effect weakens monotonically as the coefficient β increases. The physical origin of this trend lies in the fact that a large coefficient β distributes the incident energy across multiple rings, reducing the energy concentration at the high-angle components that are essential for generating the longitudinal field. Obviously, β functions as an intrinsic mode parameter that significantly influences longitudinal-field-generation efficiency in the absence of annular modulation.

When a narrowband annular modulation (∆*r* = 10% for a transmission range of 90–100%) was applied to the Bessel–Gaussian beam, the overall trend of the longitudinal field weakening with increasing the coefficient β remained unchanged. However, as shown in Figure 6, the variation becomes considerably more gradual. At NA = 0.95, the longitudinal FWHM represented only a marginal improvement compared to the circular illumination case where the FWHM decreased from 0.255λ to 0.240λ. This small difference indicates that the FWHM is relatively insensitive to β variations under annular modulation. The dominant factor for FWHM reduction is the annular modulation itself, which reduces the FWHM from 0.240λ under circular illumination to 0.197λ under this narrowband annular modulation. Moreover, this observation also suggests that this narrowband annular modulation provides a robust mechanism for focal spot compression that is tolerant to variations in the incident beam mode. Although the absolute reduction was modest, coordinated optimization of β and ring width still enabled a significant FWHM reduction from 0.240λ (Figure 5) to 0.197λ (Figure 6), achieving a sub-wavelength focusing effect beyond the diffraction limit.

### 3.5. System Mapping of Parameter Window

In addition to considering the FWHM of the longitudinal field spot, another important indicator of the longitudinal field effect is the content. To systematically evaluate the combined effects of multiple parameters, the longitudinal field content, η, is defined in Equation (6) as the ratio of longitudinal field energy to total field energy integrated within a region of a radius of 1λ:

(6) $$\eta =\frac{\int_{-\lambda }^{\lambda }{\left|E_{z}\right|}^{2}\mathrm{dS}}{\int_{-\lambda }^{\lambda }\left({\left|E_{z}\right|}^{2}+{\left|E_{r}\right|}^{2}\right)\mathrm{dS}},$$

Here, the integration range is ±λ.

**Figure 7:**
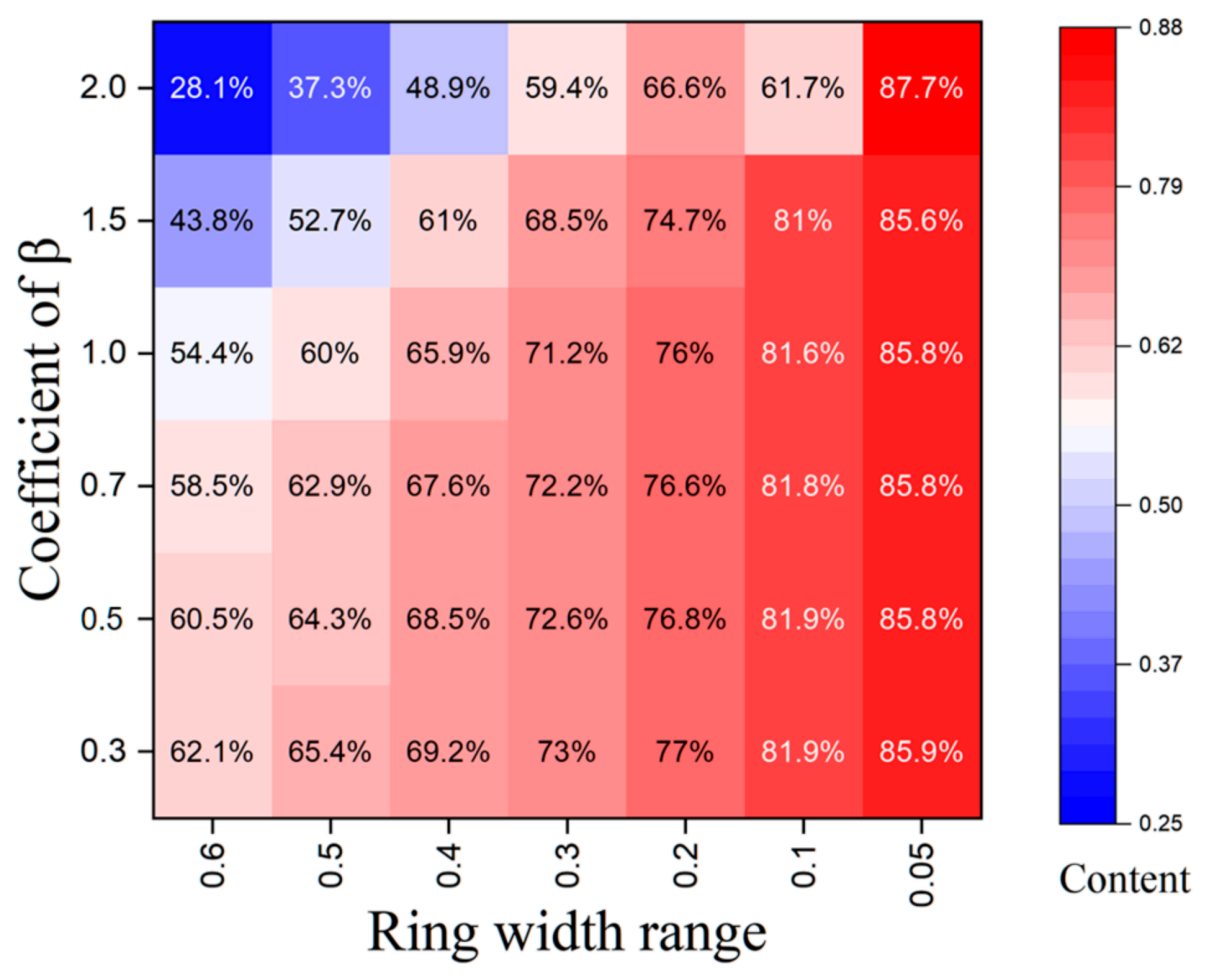
2D parameter space heat map of the longitudinal field content as a function of ring width ratio and β (NA = 0.95).

In summary, the annular modulation and high NA are key conditions for generating longitudinal field effects. Figure 7 presents a 2D heat map of the longitudinal field content as a function of ring width ratio and β at NA = 0.95. The color scale represents η, with red indicating high content and blue indicating low content. The results reveal a diagonal pattern, indicating that the ring width ratio has a more pronounced effect on the longitudinal content than β. At a ring width of 5%, the longitudinal content reached 85% across the β range from 0.3 to 2.0. Conversely, when β equals 2.0, high longitudinal content could only be achieved at extremely narrow ring widths, which imposes significant experimental challenges. For a standard Bessel–Gaussian beam with 10% annular modulation, the theoretical longitudinal content exceeds 80%.

**Figure 8:**
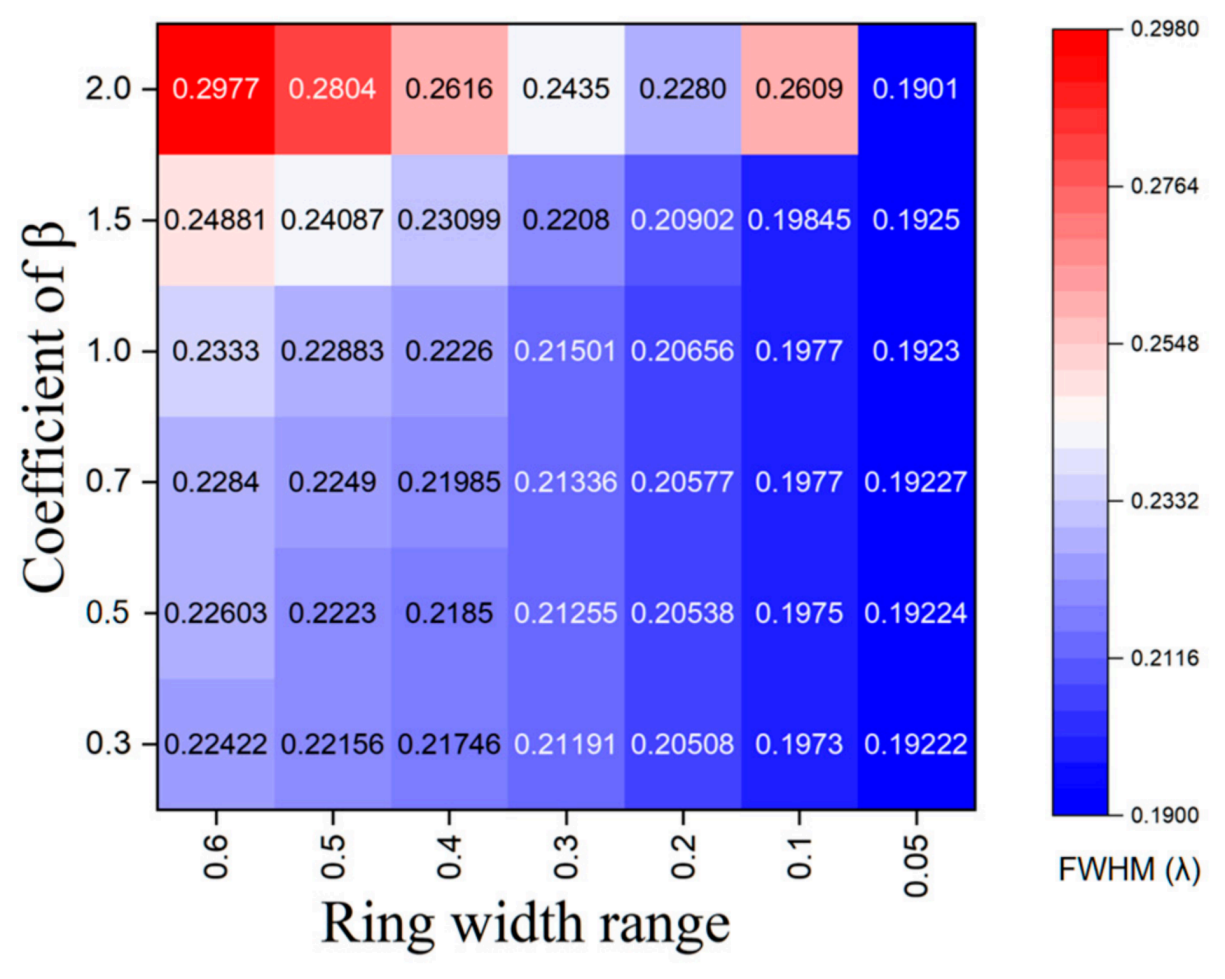
2D parameter space heat map of the longitudinal FWHM as a function of ring width ratio and *β* (NA = 0.95).

Figure 8 shows a 2D heat map of the longitudinal FWHM as a function of ring width ratio and β at NA = 0.95. The spatial mapping trend of the longitudinal spot size in the heat map is exactly opposite to the content. However, the same diagonal correlation is observed. In the weak longitudinal field regime, the FWHM reached 0.30λ with η = 28.1%; in the strong longitudinal field regime (ring width = 10%, *β* = 1.0), the FWHM decreased to 0.198λ (<0.2λ) with η = 81.6%. The maximum theoretical longitudinal content reached 87.7% under combined high-NA and narrow-band annular modulation conditions.

The underlying physical mechanism for this diagonal pattern is the trade-off between energy efficiency and spatial frequency selection. The annular aperture functions as a high-pass spatial filter that blocks low-angle components. However, when the ring becomes too narrow, the total energy passing through the aperture diminishes, leading to reduced field strength and increased sidelobes. The optimal parameter combination achieves the maximum longitudinal content while maintaining sufficient power throughput.

**Figure 9:**
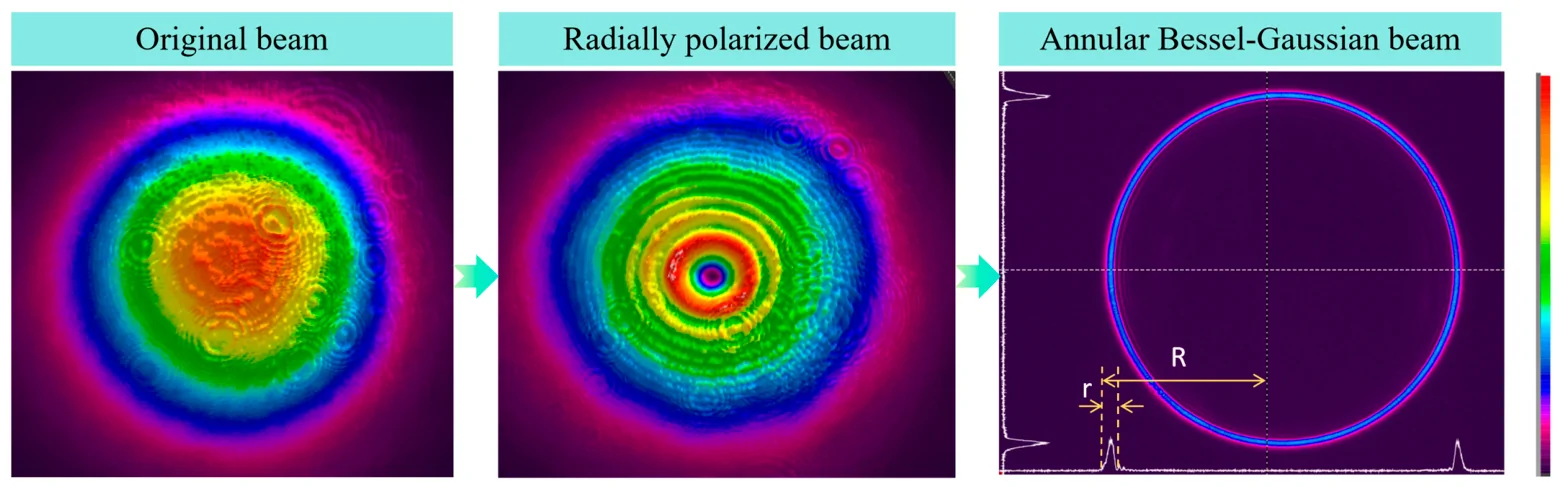
Measured beam profiles at different steps of the optical path.

For experimental validation, a beam profiler was used to record the focal spot morphology of key steps of the BGAM method. Figure 9 shows the morphology and intensity of the light spot, where colors represent energy intensity. The original incident laser is a Gaussian beam. Radial polarized beams exhibit energy hollow Bessel–Gaussian energy distribution. Based on the heat map results, the annular modulation was set to 10% (Figure 9). The annular Bessel–Gaussian modulated beam was then focused by an NA = 0.95 objective lens.

**Figure 10:**
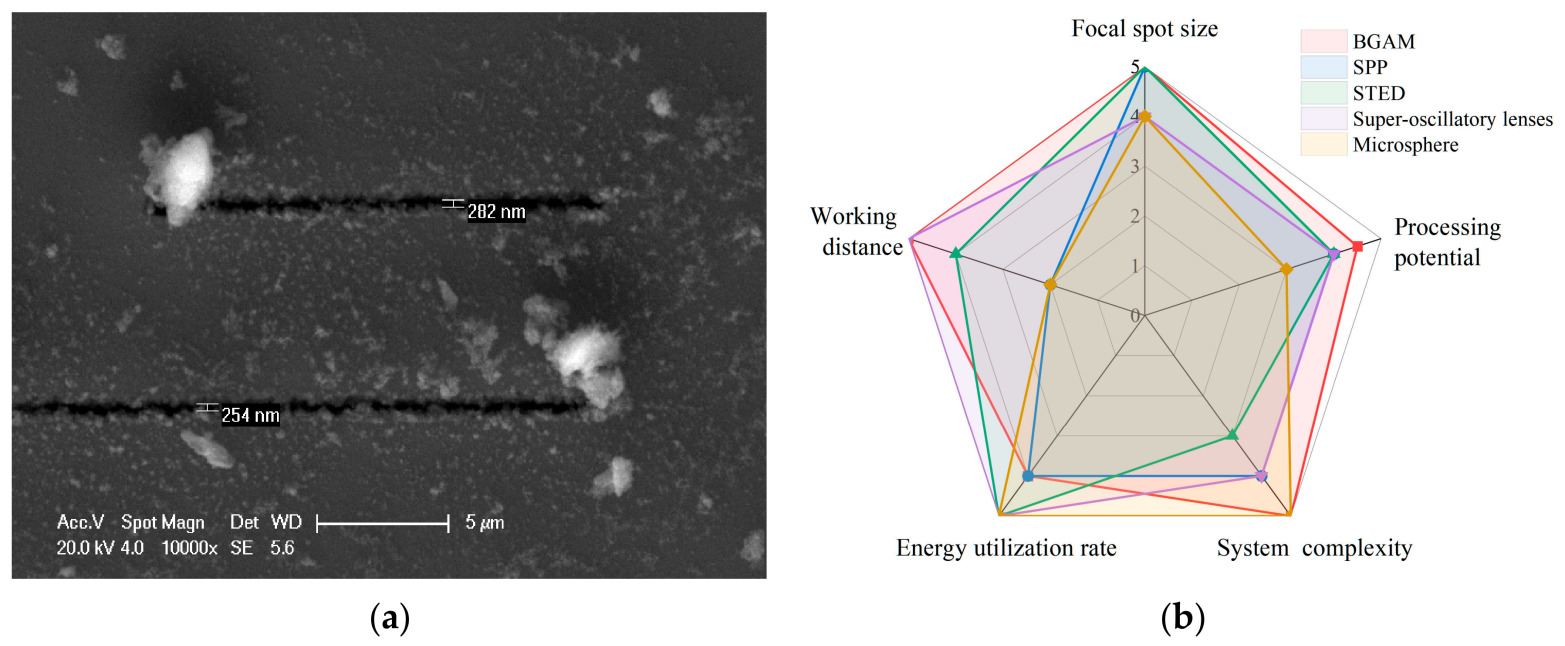
(**a**) SEM image of nanoscale scribing on a single-crystal silicon substrate. (**b**) Comprehensive performance comparison between the BGAM and other super-resolution focusing techniques.

A silicon wafer was positioned on the three-dimensional processing stage with a working distance of approximately 200 μm between the sample surface and the objective lens output port. A line scribing test was performed at a motion speed of 10 μm/s. The width of the etched groove is approximately 254 nm, corresponding to approximately 1/4 of the laser wavelength of 1033 nm (Figure 10). The measurement uncertainty of the groove width is estimated to be within ±10 nm based on the SEM resolution. The result demonstrated that the annular-modulation-enhanced longitudinal field approach has the potential for achieving sub-diffraction-limit processing (~λ/4). The unique contribution of the BGAM method lies in providing a smaller and purer longitudinal field focal spot than conventional tight focusing. Combining the BGAM method with threshold optimization to further reduce the feature size will be the subject of future work. To further clarify the advantages of the proposed BGAM method, Figure 10b presents a qualitative comparison of representative super-resolution focusing techniques, including STED, SPP, super-oscillatory lenses, microsphere and the BGAM method developed in this work [6,9,11,15,30]. The comparison is based on five dimensions: focal spot size, processing resolution, system complexity, working distance, and energy utilization rate. The BGAM method operates in the far field without near-field scanning. The longitudinal field generated can directly illuminate the sample surface from a working distance of hundreds of micrometers. This method achieves balanced performance across all five dimensions, particularly in terms of system simplicity, far-field operation, and nanomanufacturing.

## 4. Discussion

The heat map results reveal that the NA, the annular ring width ratio, and the Bessel–Gaussian coefficient β exhibit both synergistic and antagonistic effects on longitudinal field generation. The synergistic effect originates from the complementary roles of high NA and annular modulation. The antagonistic effect originates from the intrinsic ring structure of the incident beam controlled by β. When β increases beyond the optimal range, the energy distribution shifts toward higher-order rings, which reduces the effective energy within the annular aperture region. This effect partially counteracts the enhancement provided by the NA and the annular modulation. The diagonal pattern in the heat maps precisely reflects this antagonism. At larger β values, a narrower ring width is required to achieve the same longitudinal content, and the parameter window for effective longitudinal field generation becomes increasingly restricted.

Of course, the annular modulation inherently sacrifices energy throughput. When the ring width is reduced to 1%, a large fraction of the incident power is blocked. The benefit of the annular aperture is not energy recovery but spatial filtering. By blocking the low-angle components that contribute primarily to the radial field, the annular aperture enhances the relative weight of the high-angle components that dominate the longitudinal field. This improvement in longitudinal field purity and focal spot compression comes at the cost of reduced total energy. The 10% ring width represents a balance between field quality and power throughput, as further narrowing to 1% yields only marginal FWHM improvement while causing substantial energy loss.

An additional subtlety arises when β is small. When β = 0.3, the Bessel–Gaussian beam degenerates toward a quasi-Gaussian profile with energy concentrated at the center, which corresponds to low spatial frequencies. The annular modulation blocks this central region. Although the spatial match appears poor, the transmitted field originates from the edge of the transmission ring, and its angular distribution determines the longitudinal field characteristics. Consequently, the longitudinal field effect at β = 0.3 is similar to that at a β of 1.0. However, the quasi-Gaussian beam at a low β and the annular modulation are mismatched in spatial frequency, which leads to a significant reduction in energy transfer efficiency through the modulation. Therefore, the effective regulation window is bounded by the following physical constraints: (1) NA must be sufficiently high (>0.85) to satisfy the threshold for longitudinal field dominance; (2) the ring width must be optimized around 10% to balance high-frequency filtering and power throughput; and (3) *β* should be maintained below 1.5, with a preferred range of 0.7 to 1.2, to avoid excessive energy loss while preserving longitudinal field purity.

## 5. Conclusions

The core novelty of this work lies in the BGAM method that combines annular modulation and NA optimization. Radial polarization is a prerequisite input condition for generating a longitudinal electric field upon tight focusing. In summary, this work systematically investigated the generation and optimization of longitudinal electric fields through the BGAM method. Three key parameters, NA, annular ring width ratio, and the Bessel–Gaussian coefficient β, were analyzed for their individual and combined effects on the longitudinal field content and focal spot size. Annular modulation provided an effective strategy for further enhancing the longitudinal field by selectively transmitting high-angle components, which are the primary contributors to the longitudinal field. An optimal ring width of approximately 10% was identified, representing the balance between longitudinal enhancement and power throughput. The Bessel–Gaussian coefficient exhibited a monotonic weakening effect on the longitudinal field, and values below 1.5 were required to maintain longitudinal field dominance. A parameter space mapping among NA, ring width ratio, and β was established, providing quantitative guidance for selecting the optimal parameter combination. The effective regulation window was bounded by the synergistic and antagonistic interactions among these three parameters. Based on the proposed annular-modulation-enhanced method, a sub-diffraction-limit focal spot with a longitudinal field FWHM below 0.2λ was achieved. Finally, grooves with a width range of 254 nm–282 nm (approximately λ/4) were achieved, using this method on processing single-crystal silicon via a femtosecond laser of a wavelength of approximately 1033 nm under far-field conditions. This study demonstrated the potential of the proposed method for nanolithography, super-resolution imaging, and nanomanufacturing applications.

## Figures and Tables

**Table 1:** Core parameters of femtosecond laser.

Physical Quantity	Data	Physical Quantity	Data
center wavelength/nm	1033	M^2^	x: 1.13; y: 1.09
repetition frequency	up to 1 MHz	pulse width/fs	263
beam diameter/mm	2.73, 1/e^2^	polarization	linear
power stability	0.12%	beam roundness	99.2%

**Table 2:** Weight of spatial frequency influence.

θ	sin^2^θ	Contribution to the E_z_^2^ Field
≈0	≈0	≈0
*π*/4	0.5	medium
α	sin 2α	maximum

**Table 3:** Physical interpretation of the Bessel–Gaussian coefficient β in Equation (3).

β	Exponential Decay	Bessel Oscillation	Energy Distribution in θ Space
0.3	exp(−0.3x), slow decay	nearly linear	broad distribution; extends to moderate and large θ
0.5	exp(−0.5x), moderate decay	single primary ring	concentrated at moderate to large angles
0.7	exp(−0.7x), moderate decay	primary ring with weak secondary lobe	concentrated at moderate angles
1.0	exp(−x), faster decay	primary ring with distinct secondary ring	concentrated at moderate to small angles
1.5	exp(−1.5x), fast decay	multiple rings	concentrated at small angles; higher-order rings suppressed

## Data Availability

The original contributions presented in the study are included in the article, and further inquiries can be directed to the corresponding author.
